# The clinical utility of musculoskeletal ultrasonography in hemiplegic shoulder rehabilitation poststroke

**DOI:** 10.3389/fresc.2025.1576890

**Published:** 2025-05-15

**Authors:** Jun Liu, Huijuan Pan, Yong Bao, Li Huang, Yunyun Hu

**Affiliations:** ^1^ Department of Ultrasound, Ruijin Hospital, Shanghai Jiao Tong University School of Medicine, Shanghai, China; ^2^ Department of Rehabilitation Medicine, Shanghai Ruijin Rehabilitation Hospital, Shanghai, China; ^3^ Department of Ultrasound, Shanghai Ruijin Rehabilitation Hospital, Shanghai, China

**Keywords:** MSUS, shoulder pain, rehabilitation, hemiplegia, stroke

## Abstract

**Objective:**

This study aimed to assess the utility of musculoskeletal ultrasound (MSUS) in the rehabilitation of stroke patients with hemiplegic shoulder pain.

**Methods:**

We conducted a study involving 80 stroke patients with hemiplegia and concomitant shoulder pain on the affected side, admitted to our hospital between April 2020 and March 2021. MSUS was used to evaluate shoulder structures, including the long head of the biceps brachii tendon (BICT) and its sheath, rotator cuff, subacromial–subdeltoid (SA–SD) bursa, labrum, acromioclavicular ligament, acromiocoracoid ligament, and acromion–greater tuberosity (AGT) distance. We compared pre- and post-rehabilitation measurements of supraspinatus tendon (SST) thickness, BICT sheath effusion thickness, SA–SD bursa effusion thickness, AGT distance, and visual analog scale (VAS) scores. Statistical significance was set at *P* < 0.05.

**Results:**

Post-rehabilitation, the SST thickness on the hemiplegic side showed a statistically significant reduction (*P* = 0.023). No significant difference was observed in the mean maximum rupture diameter (*P* = 0.796). Both BICT sheath effusion (*P* < 0.001) and SA–SD bursa effusion (*P* < 0.001) exhibited significant decreases. The AGT distance on the hemiplegic side also demonstrated a statistically significant change (*P* < 0.001). Additionally, the VAS score significantly improved post-rehabilitation (*P* < 0.001).

**Conclusion:**

MSUS is a feasible and reproducible tool for monitoring rehabilitation progress in stroke patients with hemiplegic shoulder pain.

## 1 Introduction

Shoulder pain is a frequent complaint in stroke patients with hemiplegia, with reported incidence rates ranging from 5% to 84% (1–3). This pain often restricts both active and passive shoulder movement, impairing joint mobility and ultimately hindering rehabilitation progress, thereby delaying the recovery of upper limb function (4). The pathophysiology of poststroke shoulder injury is complex, primarily attributed to impaired motor control and alterations in peripheral and central nervous system activity **(**5). Contributing factors include glenohumeral subluxation (GHS) and various soft tissue injuries (STIs), such as effusion of the long head of the biceps brachii tendon (BICT) sheath, rotator cuff lesions, and subacromial–subdeltoid (SA–SD) bursal effusion (6).

Musculoskeletal ultrasound (MSUS) offers distinct advantages in diagnosing shoulder disorders and periarticular soft tissue pathologies by providing a clear visualization of soft tissue lesion extent, morphological changes, and vascular distribution patterns (7). Unlike other imaging modalities, MSUS enables dynamic assessment of musculoskeletal structures during movement, facilitating the detection of subtle abnormalities that may remain undetected during static examination (7). Furthermore, MSUS permits frequent repeat examinations, allowing for dynamic, multiplanar evaluation of pathological progression. Comparative analysis with contralateral healthy structures enhances diagnostic accuracy, with studies demonstrating comparable specificity to magnetic resonance imaging (MRI) for certain indications (8).

In poststroke hemiplegic shoulder pain, where soft tissue injuries (STIs) and glenohumeral subluxation (GHS) frequently coexist, clinicians require practical imaging tools to guide rehabilitation strategies. While MRI remains the gold standard, MSUS presents a viable alternative for monitoring rehabilitation outcomes. This study investigates whether MSUS can provide objective indicators correlating with subjective pain improvement, thereby validating patient-reported outcomes. We hypothesize that MSUS may serve as a feasible imaging modality for (1) evaluating rehabilitation efficacy, (2) identifying objective markers of clinical improvement, and (3) informing rehabilitation program adjustments. Our findings aim to establish the clinical utility of MSUS in managing hemiplegic shoulder pain during stroke rehabilitation.

## 2 Materials and methods

### 2.1 Patients

#### 2.1.1 Sample size determination

The sample size was calculated using the standard formula for paired quantitative data studies:

$$n\;=\;[(Z\alpha /2\;+\;Z\beta {)}^{2}\;\times \;\sigma^{2}]\;/\;\delta^{2}$$

Where:

• *n* = required sample size per group
• Z*α*/2 = 1.96 (two-tailed, *α* = 0.05)
• Z*β* = 1.28 (power = 90%, *β* = 0.1)
• *σ* = 1.3 (standard deviation from pilot data)
• *δ* = 0.5 (clinically meaningful difference)

Accounting for an anticipated 10% dropout rate, the final sample size was determined to be 80 participants.

#### 2.1.2 Study population

This study retrospectively analyzed 80 stroke patients with hemiplegic shoulder pain who underwent MSUS evaluation at our institution between April 2020 and March 2021. Age- and sex-matched stroke patients without shoulder pain served as controls.

#### 2.1.3 Ethical considerations

This study was conducted in accordance with the:

1. Declaration of Helsinki (1964) and its subsequent amendments
2. Institutional review board approval (include approval number if available)
3. National research ethics guidelines

Written informed consent was obtained from all participants prior to study enrollment.

#### 2.1.4 Inclusion criteria

Participants were included based on the following criteria:

1. Diagnosis of first-ever stroke confirmed by CT or MRI according to standardized cerebrovascular disease criteria
2. Presence of hemiplegia with unilateral upper limb involvement and concomitant shoulder pain
3. Absence of pre-existing shoulder pain or prior shoulder surgery
4. Medically stable condition poststroke with:
  (1) Stable vital signs
  (2) Clear consciousness
  (3) Ability to comply with complete examination protocols

#### 2.1.5 Exclusion criteria

Patients were excluded if they met any of the following conditions:

1. History of shoulder dysfunction or prior shoulder pathology
2. Comorbid myopathic disorders or peripheral nervous system diseases
3. Inability to maintain a seated position independently or with minimal assistance (defined as requiring >1 person for support).

### 2.2 Equipment and methods

Musculoskeletal ultrasound examinations were performed using a high-resolution ultrasound system (LOGIQ E9, GE Healthcare, Chicago, IL, USA) equipped with a linear-array transducer (frequency range, 9–16 MHz). The system's preset musculoskeletal imaging parameters were optimized for shoulder evaluation, including appropriate depth settings (3–5 cm) and focal zone adjustments to ensure optimal visualization of superficial and deep shoulder structures.

### 2.3 Examination protocol

#### 2.3.1 Soft tissue injury (STI) assessment

Ultrasonographic evaluation of shoulder structures was performed according to standardized protocols established by Martinoli (9), with the following specific assessment criteria:

1. **Biceps brachii tendon (BICT) and sheath:**
  (1) Tendon morphology: assessment for abnormal thickening, focal hypo-/hyper-echoic regions, or structural discontinuity
  (2) Sheath evaluation: presence of pathological effusion (>2 mm in width)
2. Rotator cuff components:

Systematic evaluation of all four tendons (SUBT, SST, INFT, TMT) for:

(1) Morphological abnormalities (thickening)
(2) Focal echo texture alterations (hypo-/hyper-echoic regions)
(3) Structural integrity (tears or defects)

(3) **Subacromial–subdeltoid (SA–SD) bursa:**

Quantitative assessment of bursal effusion (>2 mm threshold)

(4) **Glenoid labrum:**

Evaluation for pathological fluid accumulation in both anterior and posterior regions.

(5) **Ligamentous structures:**

Acromioclavicular and coracoclavicular ligaments assessed for:

(1) Structural injury (tears)
(2) Functional integrity (laxity)

#### 2.3.2 Assessment of glenohumeral subluxation (GHS)

GHS was quantitatively evaluated using the standardized ultrasound measurement protocol described by Kumar et al. (10). The examination procedure included:

1. Acromion–greater tuberosity distance (AGT):
  (1) Measurement of the maximal perpendicular distance between the lateral border of the acromion and the apex of the greater tuberosity
  (2) Bilateral assessment (affected vs. unaffected side) for comparative analysis
  (3) Measurements obtained in standardized shoulder position (specify if applicable, e.g., neutral position with arm adducted)
2. Measurement protocol:
  (1) Three consecutive measurements performed to ensure reliability
  (2) Mean value used for final analysis
  (3) All measurements conducted by experienced sonographers using identical transducer positioning

### 2.4 Pain assessment using visual analog scale (VAS)

The VAS was employed to quantify pain intensity according to standardized protocols. The assessment methodology comprised:

#### 2.4.1 VAS administration protocol

1. A 10 cm horizontal line was presented to patients, anchored with:
  (1) 0 cm (left terminus): “no pain”
  (2) 10 cm (right terminus): “worst imaginable pain”
2. Patients were instructed to mark their current pain level on the line.
3. Measurements were recorded to the nearest millimeter using a calibrated ruler.

#### 2.4.2 Scoring interpretation

Pain severity was categorized as follows:

1. 0: pain-free
2. 1–3: mild, intermittent pain (does not interfere with daily activities)
3. 4–5: moderate pain (tolerable but affecting sleep quality)
4. 6–7: substantial pain (frequent, significantly impairing sleep)
5. 8–9: severe pain (persistent, causing sleep deprivation)
6. 10: excruciating pain (completely debilitating)

#### 2.4.3 Clinical classification

Following Lindgren's criteria:

1. VAS ≥ 4: diagnosed as hemiplegic shoulder pain
2. VAS < 4: classified as non-hemiplegic shoulder pain

### 2.5 Examination protocol

#### 2.5.1 Sonographic examination procedures

All MSUS examinations were performed by an experienced sonographer (>5 years of musculoskeletal specialization) who was blinded to patient clinical status. The standardized protocol was adapted from Martinoli's (9) shoulder examination methodology, with the following implementation details:

1. Patient positioning:
  (1) Seated position facing the examiner
  (2) Upper limbs maintained in standardized positions by an assistant
  (3) Bilateral examination (hemiplegic and unaffected sides)
2. Systematic scanning protocol:

Sequential evaluation of:

(1) BICT and sheath
(2) Rotator cuff tendons (SUBT, SST, INFT, TMT)
(3) Glenoid labrum
(4) Acromioclavicular and coracoclavicular ligaments
(5) Subacromial–subdeltoid (SA–SD) bursa

#### 2.5.2 Pre-rehabilitation assessment

1. SST evaluation:
  (1) Thickness measurement (hemiplegic vs. unaffected side)
  (2) Maximum tear diameter quantification
2. Effusion assessment:
  (1) BICT sheath effusion thickness
  (2) SA–SD bursal effusion thickness
3. Glenohumeral stability:
  (1) Acromion–greater tuberosity (AGT) distance measurement
  (2) Comparative analysis of GHS (hemiplegic vs. unaffected side)
4. Pain quantification:
  (1) VAS scoring for hemiplegic shoulder pain

#### 2.5.3 Post-rehabilitation assessment

Identical protocol performed following rehabilitation intervention, with additional comparative analyses:

1. Longitudinal comparison of all parameters (pre- vs. post-rehabilitation)
2. Maintained contralateral comparison (hemiplegic vs. unaffected side)

### 2.6 Rehabilitation treatment

A comprehensive rehabilitation protocol was implemented, consisting of programmed manual therapy, extracorporeal shockwave therapy (ESWT), kinesio taping (KT), and orthotic intervention.

1. **Programmed manual therapy**: This intervention included muscle tension reduction, joint range-of-motion training, soft tissue mobilization, and joint mobilization. The primary objectives were to decrease muscle tone and improve joint mobility. Muscle tension reduction and joint motion training were administered twice daily, while soft tissue and joint mobilization were performed once daily.
2. **ESWT**: Treatment was delivered using a 15 mm probe at an impact frequency of 6–10 Hz and a pressure of 1.5–3.0 bar, with 2,000 shocks per session. Sessions were conducted twice weekly.
3. **KT**: Type I KT was applied with the anchor fixed at the superior aspect of the humeral greater tubercle and terminated at the scapular supraspinatus fossa. Each application remained in place for 48 h, followed by a 24 h rest period before reapplication.
4. **Orthotic intervention**: A dual-support orthosis was utilized to stabilize both proximal and distal segments. The device was designed to offload arm weight, correct glenohumeral subluxation, and minimize passive shoulder movement. Orthotic use was gradually tapered rather than abruptly discontinued, with daily wear time progressively reduced. However, the orthosis was reapplied if pain recurred.

The total duration of rehabilitation was 12 weeks for all interventions.

### 2.7 Statistical analysis

All statistical analyses were performed using SPSS 25.0 (IBM Corp., Armonk, NY, USA). Continuous data are presented as mean ± standard deviation (SD). To evaluate the effects of rehabilitation across time (pre- vs. post-rehabilitation) and between groups (hemiplegic vs. unaffected side), a two-way repeated-measures analysis of variance (ANOVA) was conducted with **time** (pre, post) and **group** (hemiplegic, unaffected) as within-subject factors. The interaction effect (time × group) was included in the model to assess whether changes over time differed between groups. For significant interaction effects, *post hoc* pairwise comparisons were performed using paired *t*-tests with Bonferroni correction to control for Type I error. A two-tailed *P*-value of <0.05 was considered statistically significant.

## 3 Results

The study included 80 stroke patients (47 males, 33 females) aged 52–82 years (mean, 72 ± 7.68 years). The mean poststroke duration was 2.3 months (range, 1–3 months). The cohort comprised 65 cases of cerebral infarction and 15 cases of cerebral hemorrhage. Hemiplegic shoulder involvement included 45 left-sided and 35 right-sided cases.

All patients exhibited shoulder disorders on the affected side, confirmed by MSUS, with concomitant shoulder pain (VAS score ≥4) (Table 1).

**Table 1:** The demographic data for all the patients.

Patients	Gender		Age	Stroke		Hemiplegia shoulder	
	Male	Female		Cerebral infarction	Brain blood	Right	Left
80	47	33	72 ± 7.68	65	15	35	45

### 3.1 SST findings

MSUS revealed abnormalities exclusively in the SST (11), with no pathological changes observed in the subscapularis (SUBT), infraspinatus (INFT), or teres minor (TMT) tendons.

#### 3.1.1 SST thickness

**Figure 1:**
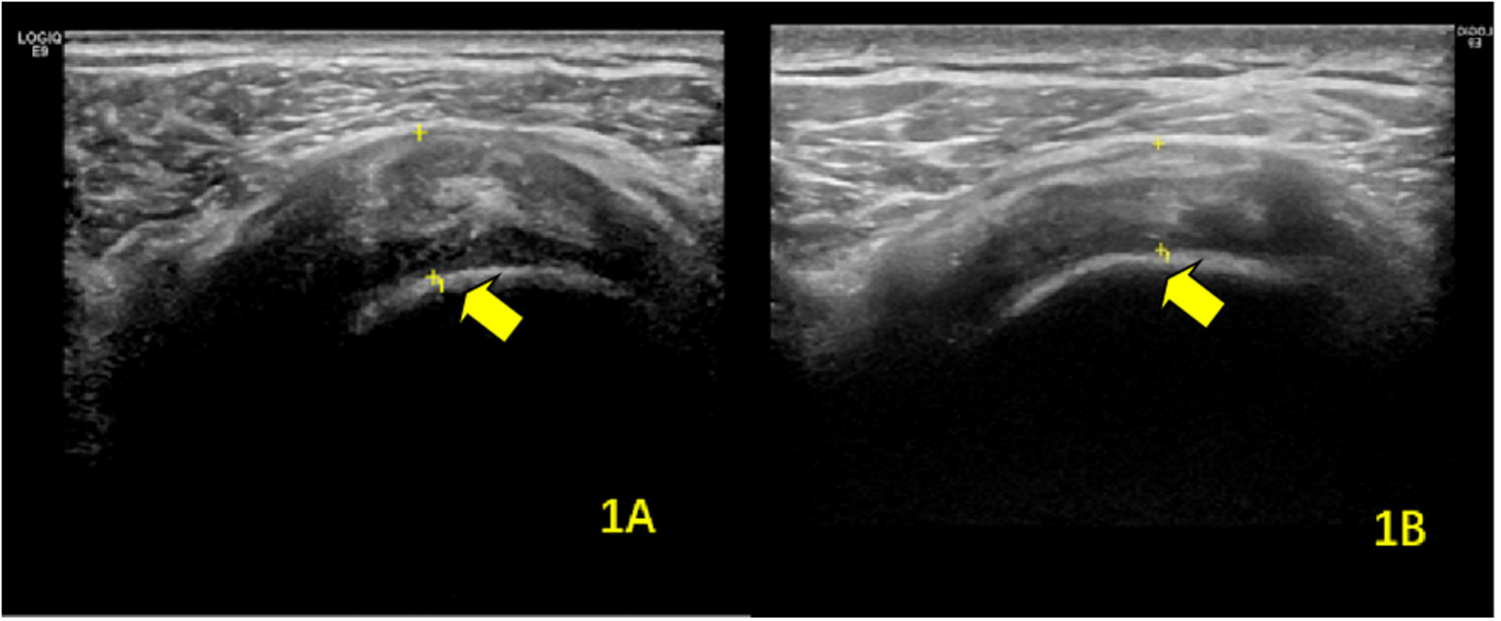
Ultrasonographic findings of supraspinatus tendinitis in the hemiplegic shoulder **(A)** before and **(B)** after rehabilitation therapy. The arrows indicate the affected supraspinatus tendon.

Repeated-measures ANOVA revealed significant main effects of **time** (*F*_(1,79)_ = 18.32, *P* < 0.001) and **group** (*F*_(1,79)_ = 145.67, *P* < 0.001), as well as a significant **time** **×** **group** interaction (*F*_(1,79)_ = 6.89, *P* = 0.011). *Post hoc* analysis demonstrated a significant reduction in SST thickness on the hemiplegic side post-rehabilitation (8.46 ± 0.67 mm vs. 8.23 ± 0.36 mm; *P* = 0.023), whereas no significant change was observed on the unaffected side (6.54 ± 0.59 mm vs. 6.60 ± 0.42 mm; *P* = 0.462) (data presented in Table 2; Figure 1).

**Table 2:** Repeated-measures ANOVA results for SST thickness.

Factor	*F*-value (df)	*P*-value	*Post hoc* comparisons (Bonferroni-adjusted)
Time	18.32 (1,79)	<0.001	Pre vs post (Hemiplegic): *P* = 0.023
Group	145.67 (1,79)	<0.001	Hemiplegic vs unaffected (pre): *P* < 0.001
Time × group	6.89 (1,79)	0.011	Hemiplegic vs unaffected (post): *P* < 0.001

#### 3.1.2 Tendon tears

All identified tears were localized to the SST. The mean maximum tear diameter showed no significant change following rehabilitation (pre, 4.34 ± 0.47 mm; post, 4.56 ± 0.41 mm; *P* = 0.796).

### 3.2 BICT findings

#### 3.2.1 Pre-rehabilitation findings

Among the 80 patients, 44 (55%) exhibited biceps tenosynovitis (BICT involvement), which presented as:

1. Isolated tendon sheath effusion (>2 mm) in 32 cases (mean thickness, 2.98 ± 0.61 mm)
2. Tenosynovitis with concomitant effusion in 12 cases

#### 3.2.2 Post-rehabilitation outcomes

The prevalence of BICT pathology decreased to 18 cases (22.5%), consisting of:

1. Eight cases with isolated sheath effusion (>2 mm)
2. Ten cases demonstrating tenosynovitis with effusion

**Figure 2:**
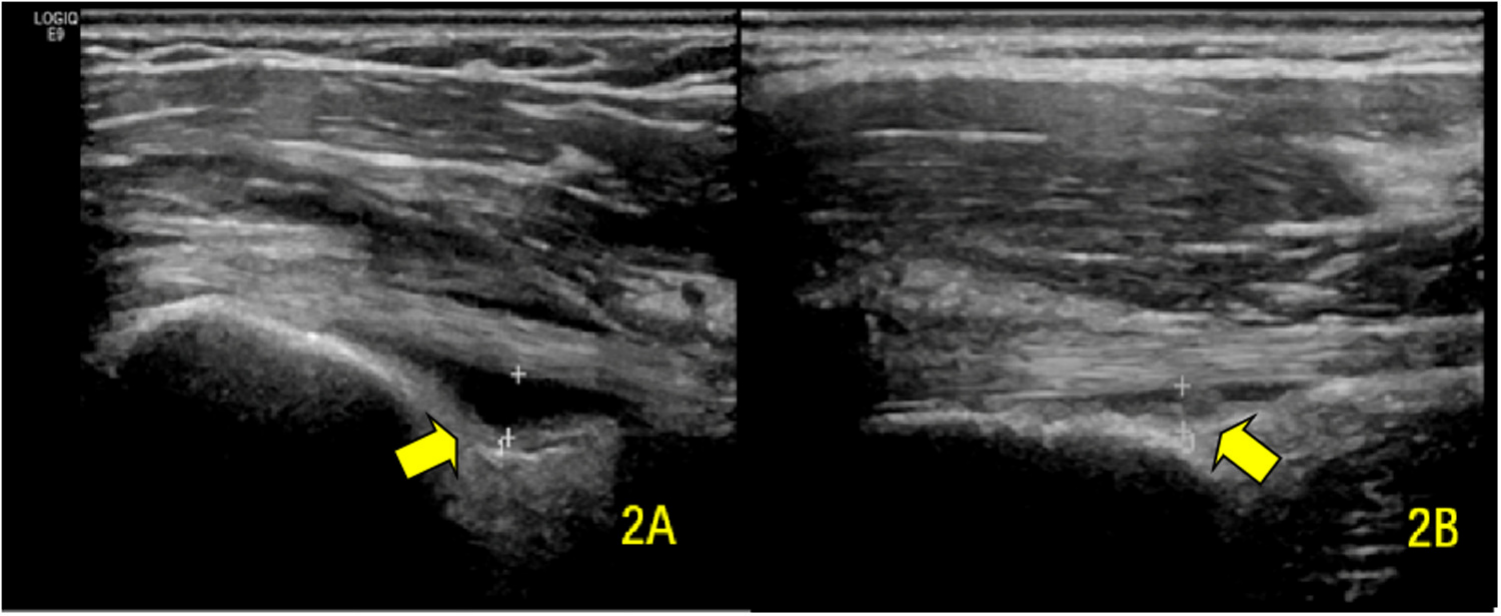
Transverse ultrasound images showing BICT sheath effusion **(A)** pre-rehabilitation and **(B)** post-rehabilitation. The arrows demarcate the fluid-distended BICT synovial sheath.

In the subgroup with initial effusion (*n* = 32), post-treatment measurements showed a significant reduction in sheath thickness (1.82 ± 0.45 mm vs. baseline; *P* < 0.001) (Figure 2).

### 3.3 SA–SD bursa findings

#### 3.3.1 Pre-rehabilitation assessment

Ultrasound examination identified SA–SD bursal effusion (>2 mm) in 21 of 80 patients (26.3%), with a mean effusion thickness of 2.55 ± 0.34 mm.

#### 3.3.2 Post-rehabilitation outcomes

**Figure 3:**
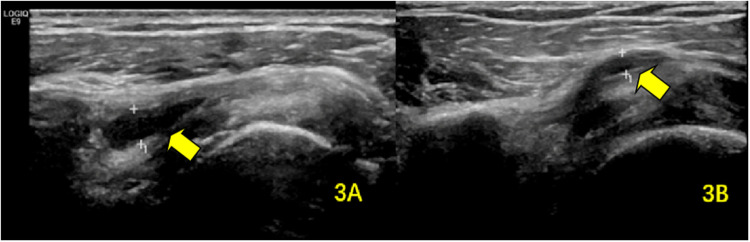
SA–SD bursal effusion demonstrated on ultrasonography **(A)** at baseline and **(B)** following rehabilitation. The arrows highlight the pathological fluid accumulation within the SA–SD bursa.

The prevalence of bursal effusion decreased significantly to nine cases (11.3%). Among the initial 21 patients with effusion, post-treatment measurements demonstrated a marked reduction in mean thickness (1.12 ± 0.64 mm vs. baseline; *P* < 0.001) (Table 3; Figure 3).

**Table 3:** Comparison of the maximum value of rupture, BICT, and SA–SD bursal before and after rehabilitation therapy.

Rehabilitation therapy	Average maximum value of SST rupture (mm)	Thickness of BICT sheath effusion (mm)	Thickness of SA–SD bursal effusion (mm)
Before	4.34 ± 0.47	2.98 ± 0.61 (44/80)	2.55 ± 0.34 (21/80)
After	4.56 ± 0.41	1.82 ± 0.45 (44/80)	1.12 ± 0.64 (21/80)
*P*-value	>0.05	<0.01	<0.01

### 3.4 GHS findings

**Figure 4:**
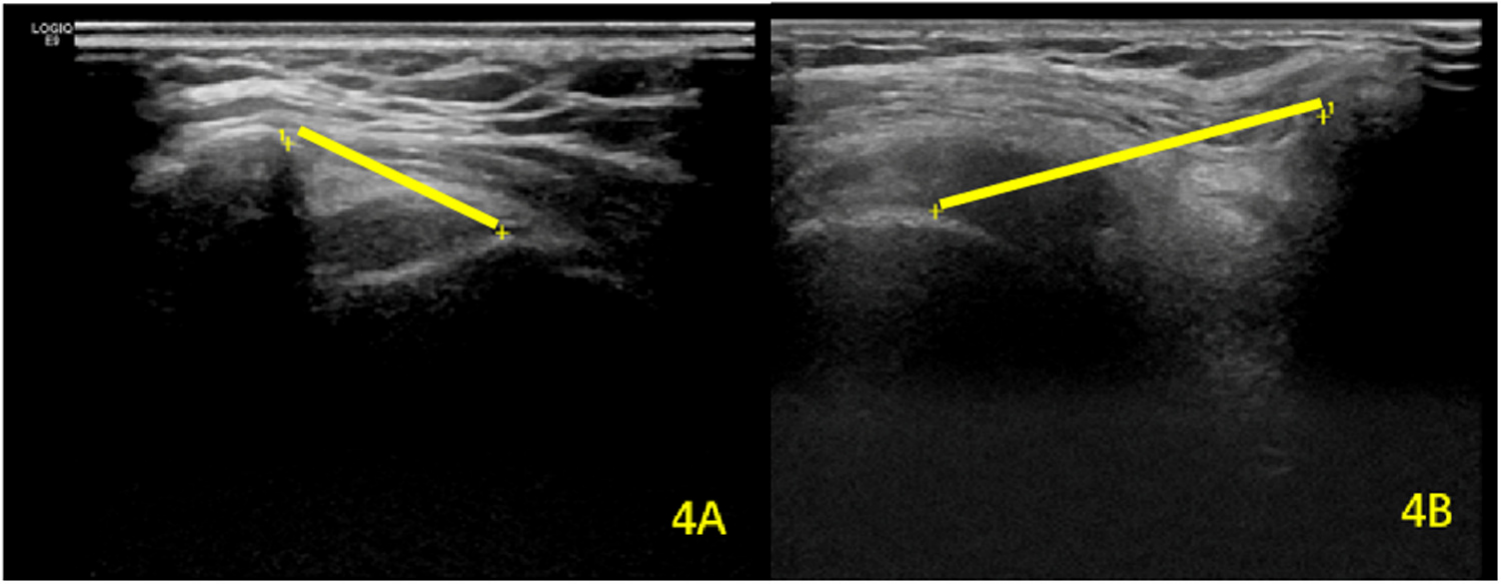
Comparative analysis of AGT distance between **(A)** the unaffected contralateral side and **(B)** the hemiplegic shoulder. The yellow straight lines represent the measured AGT interval.

For AGT distance, repeated-measures ANOVA showed significant main effects of time (*F*_(1,79)_ = 32.15, *P* < 0.001) and group (*F*_(1,79)_ = 89.43, *P* < 0.001), with a significant time × group interaction (*F*_(1,79)_ = 12.76, *P* < 0.001). *Post hoc* comparisons confirmed a significant decrease in AGT distance on the hemiplegic side post-rehabilitation (25.54 ± 5.32 mm vs. 21.48 ± 3.79 mm; *P* < 0.001), while the unaffected side remained stable (17.36 ± 3.35 mm vs. 16.76 ± 3.76 mm; *P* = 0.816) (Table 4; Figure 4).

**Table 4:** Repeated-measures ANOVA results for AGT distance.

Factor	*F*-value (df)	*P*-value	*Post Hoc* comparisons (Bonferroni-adjusted)
Time	32.15 (1,79)	<0.001	Pre vs post (hemiplegic): *P* < 0.001
Group	89.43 (1,79)	<0.001	Hemiplegic vs unaffected (pre): *P* < 0.001
Time × group	12.76 (1,79)	<0.001	Hemiplegic vs unaffected (post): *P* < 0.001

### 3.5 VAS pain scores

#### 3.5.1 Pre-rehabilitation distribution

The baseline VAS scores demonstrated moderate-to-severe pain among participants:

1. 4–5 points: 48 patients (60%)
2. 6–7 points: 22 patients (27.5%)
3. 8–9 points: 8 patients (10%)
4. 10 points: 2 patients (2.5%)

No patients reported mild pain (1–3 points) or absence of pain (0 points).

#### 3.5.2 Post-rehabilitation outcomes

Following treatment, we observed significant pain reduction:

1. 1–3 points (mild pain): 54 patients (67.5%)
2. 4–5 points: 22 patients (27.5%)
3. 6–7 points: 2 patients (2.5%)
4. 8–9 points: 2 patients (2.5%)

No patients reported severe pain (10 points).

The intervention resulted in a statistically significant VAS score reduction (*P* < 0.001) (Table 5).

**Table 5:** Comparison of SA-SD bursal before and after rehabilitation therapy.

Rehabilitation therapy	Bursal effusion (case)	Thickness of effusion (mm)
Before	21	2.55 ± 0.34 (21/80)
After	9	1.12 ± 0.64 (21/80)
*P* value		0.000

## 4 Discussion

The increasing global incidence of stroke in recent years has brought heightened attention to poststroke rehabilitation strategies (12). As an integral component of comprehensive stroke management, rehabilitation therapy has been well-established as the most effective approach for reducing disability and improving functional outcomes (1–4). A frequently observed complication during rehabilitation is the acute onset of shoulder pain, which often presents as a clinically significant symptom requiring prompt medical attention. This pain manifestation not only compromises patient comfort but also substantially impedes the rehabilitation process. Current evidence indicates that STIs and GHS represent two primary etiological factors contributing to this debilitating pain syndrome in poststroke patients (6). The pathophysiological mechanisms underlying these conditions involve complex interactions between neuromuscular impairment, biomechanical stress, and inflammatory responses, which collectively exacerbate shoulder dysfunction and hinder recovery progress. This study aimed to assess the clinical utility of musculoskeletal ultrasound in monitoring rehabilitation outcomes among stroke patients with hemiplegic shoulder pain. Our findings demonstrate that musculoskeletal ultrasound represents a reliable and reproducible modality for tracking rehabilitation progress in this patient population.

The pathogenesis of STIs in poststroke patients involves a multifactorial interplay between intrinsic and extrinsic factors (13). Intrinsic predisposing factors primarily consist of diminished neuromuscular activation, compromised biomechanical properties of musculoskeletal tissues, and impaired vascular perfusion. Extrinsic contributors encompass traumatic insults, inappropriate therapeutic exercise regimens, and iatrogenic surgical complications. This vulnerability is particularly pronounced in elderly stroke populations due to age-related degenerative changes in tendon structure and function, which markedly reduce tissue resilience. The pathophysiological cascade is further exacerbated by characteristic poststroke musculoskeletal alterations, including restricted glenohumeral mobility, abnormal scapulohumeral kinematics, and impaired movement patterns. These pathological changes render both active and passive rehabilitation maneuvers potentially injurious, frequently precipitating STIs. The resultant shoulder pain establishes a vicious cycle by progressively limiting the joint range of motion, compromising functional recovery, and ultimately undermining rehabilitation outcomes. This complex pathophysiology underscores the need for carefully tailored rehabilitation approaches in this vulnerable patient population.

The development of STIs in poststroke patients primarily results from neuromuscular dysfunction during both the early hypotonic and late hypertonic phases following cerebral hemorrhage or infarction. During these phases, rotator cuff muscles exhibit significantly reduced activation due to impaired neural innervation, while simultaneously being subjected to continuous mechanical stress from humeral head displacement. This pathophysiological state leads to sustained tensile loading of the SST, SUBT, and BICT, precipitating a sterile inflammatory cascade characterized by initial edema and synovial effusion, followed by progressive tendon degeneration marked by fibrosis, reduced elasticity, and increased susceptibility to partial or complete rupture (14).

Our imaging findings revealed several clinically significant observations: First, we documented substantial SST thickening on the hemiplegic side compared with the unaffected side at baseline (*P* < 0.001), with post-rehabilitation measurements showing significant reduction (*P* = 0.023), though persistent inter-limb differences remained (*P* < 0.001). This residual asymmetry suggests the chronic nature of rotator cuff pathology may necessitate extended rehabilitation beyond our 12-week intervention period. Second, while we observed no SUBT abnormalities—contrasting with Murie-Fernandez et al.'s report **(**15)—our findings regarding SST improvements aligned with their therapeutic outcomes **(**15). Third, rehabilitation produced marked reductions in both the prevalence and severity of BICT tenosynovitis (*P* < 0.001) and SA–SD bursal effusions (*P* < 0.001), indicating our protocol effectively addressed inflammatory components of STI. These improvements in tendinopathy and effusion parameters, consistent with established literature (16), demonstrate the efficacy of our rehabilitation approach in modifying the pathological progression of hemiplegic shoulder pain.

GHS represents a frequent complication of poststroke hemiplegia, with an estimated incidence of 30%–80% in affected patients (17). This condition predominantly develops during the early hypotonic phase following stroke, when the profound weakness of the shoulder girdle musculature fails to counteract the gravitational pull on the affected upper limb, resulting in inferior displacement of the humeral head relative to the glenoid fossa. The resultant malalignment creates excessive traction on periarticular structures, including the subacromial bursa and rotator cuff neural elements, leading to mechanical irritation and subsequent soft tissue damage that manifests clinically as shoulder pain (18). Without timely intervention, this pathological process may progress to a cascade of secondary complications including chronic shoulder pain, STIs, and shoulder–hand syndrome, ultimately compromising upper limb functional recovery and impairing activities of daily living (19, 20).

Current clinical assessment of GHS primarily relies on two modalities: physical palpation and radiographic imaging. While palpation offers the advantage of being readily accessible and inexpensive, this technique suffers from significant limitations including operator-dependent subjectivity, limited accuracy (particularly for mild subluxation), and poor sensitivity for early-stage detection (21). Conventional radiography, although providing objective measurements, presents practical challenges in rehabilitation settings due to repeated radiation exposure and poor suitability for serial monitoring. In contrast, MSUS has emerged as an ideal imaging modality for GHS evaluation, offering three distinct advantages: (1) complete absence of ionizing radiation, making it safe for repeated assessments; (2) capacity for real-time, dynamic evaluation without temporal restrictions; and (3) simultaneous visualization of periarticular soft tissues, enabling comprehensive analysis of contributing factors to GHS pathogenesis. These characteristics make MSUS particularly valuable for both diagnostic evaluation and therapeutic monitoring in the rehabilitation setting.

MSUS has emerged as a reliable imaging modality for quantifying GHS in clinical practice. Pioneering work by Park et al. (22) established the excellent test–retest reliability of MSUS measurements for AGT distance in poststroke hemiplegia, a finding subsequently corroborated by Kumar et al. (23) who further demonstrated its minimal measurement error in clinical applications. In our cohort study, MSUS evaluation revealed significant GHS in all 80 hemiplegic patients at baseline, with the affected side demonstrating markedly greater AGT distances compared with the unaffected side (*P* < 0.001). Post-rehabilitation assessments showed substantial improvement in GHS parameters, evidenced by significant reductions in AGT measurements on the hemiplegic side (*P* < 0.001), consistent with the therapeutic outcomes reported by Türkkan et al. (24). However, unlike Türkkan's findings, persistent inter-side differences remained after treatment (*P* < 0.001), potentially attributable to the relatively short 12-week intervention period in our study. Importantly, our results confirm that MSUS maintains excellent reliability and discriminant validity for serial AGT measurements throughout the rehabilitation process, making it particularly suitable for both initial assessment and longitudinal monitoring of GHS progression in poststroke patients.

VAS serves as a well-validated instrument for quantitative pain assessment in clinical research (25). Our findings demonstrated a statistically significant reduction in VAS scores following rehabilitation intervention (*P* < 0.001), indicating substantial improvement in patients’ subjective pain experience. This clinical improvement was corroborated by objective ultrasonographic measures, including (1) decreased SST thickness; (2) stabilization of pre-existing tendon tears without further expansion; (3) reduction in BICT sheath effusion; (4) diminished SA–SD bursal effusion; and (5) decreased AGT distance. The concordance between these objective imaging parameters and patients’ subjective pain relief provides robust evidence for the therapeutic efficacy of our rehabilitation protocol, validating both its structural and functional benefits.

In conclusion, MSUS represents a feasible imaging alternative to MRI for evaluating STIs and GHS in poststroke hemiplegic patients with shoulder involvement. As a readily accessible imaging modality, MSUS offers distinct advantages in accurately identifying pathological changes in the hemiplegic shoulder while enabling serial examinations to objectively monitor rehabilitation progress. This retrospective study demonstrates significant correlations between ultrasonographic improvements (including reduced tendon thickness, effusion volumes, and AGT distances) and pain reduction as measured by VAS scores. However, the relative contribution of each parameter to clinical improvement warrants further investigation through prospective studies. Additionally, future research should incorporate extended rehabilitation durations to address the temporal limitations identified in our current protocol, thereby providing more comprehensive insights into the long-term therapeutic outcomes.

## Data Availability

The original contributions presented in the study are included in the article/Supplementary Material, further inquiries can be directed to the corresponding author.
